# Mid-pregnancy circulating cytokine levels, placental efficiency and their relationship with preterm birth

**DOI:** 10.61622/rbgo/2024rbgo58

**Published:** 2024-07-26

**Authors:** Carlos Grandi, Karina Bezerra Salomão, Stella Felippe de Freitas, Paulo Ricardo Higassiaraguti Rocha, Ricardo de Carvalho Cavalli, Viviane Cunha Cardoso

**Affiliations:** 1 Argentine Society of Pediatrics Buenos Aires Argentina Argentine Society of Pediatrics, Ciudad Autónoma de Buenos Aires, Buenos Aires, Argentina.; 2 Faculty of Medicine of Ribeirão Preto Universidade de São Paulo Ribeirão Preto SP Brazil Faculty of Medicine of Ribeirão Preto, Universidade de São Paulo, Ribeirão Preto, SP, Brazil.

**Keywords:** Cytokines, Preterm birth, Pregnancy, Placenta

## Abstract

**Objective:**

To assess a panel of cytokines and placental insufficiency with the risk of preterm delivery (PTD).

**Methods:**

Nested case-control study into the BRISA birth cohort. Eighty-two mother-infant-placenta pairs were selected at 20^+0^ to 25^+6^ weeks. Circulating biomarker levels were performed using Luminex flowmetric xMAP technology. Cytokines classified as Th1, Th2 or Th17 and other biomarkers were selected. The ratio between birth weight and placental weight (BW/PW) was used as a proxy for placental efficiency. Spearman correlation, univariate analyses and logistic regression models were calculated. Sensitivity, specificity, positive and negative likelihood ratios were calculated using the Receiver Operating Characteristic curve.

**Results:**

Mean gestational age was 250 days, 14,6% were small for gestational age, 4,8% large for gestational age and 13,4% stunted. Placental efficiency was higher for term newborns (p<0,001), and 18/22 (81%) preterm biomarker values were higher than the control group. Th1 cytokines were highly correlated, while the weakest correlation was observed in other biomarkers. Less education was associated with a higher risk of PTD (p = 0.046), while there was no appreciable difference in the risk of PTD for placental insufficiency. Biomarkers showed negligible adjusted OR of PTD (0.90 to 1.02). IL-6, IL-8, IL-1β, TNFβ, IL-4, IL-13, GCSF, MIP1A, VEGF, EGF, and FGF2 presented a higher sensitivity ranging from 75.56% to 91.11%.

**Conclusion:**

IL-8, IL-12p40, IL-4, IL-13, GCSF, MIP1B, and GMSF in asymptomatic pregnant women were associated with PTD. This finding suggests an activation of maternal inflammatory response.

## Introduction

In 2020, there were an estimated 13.4 million preterm livebirths, constituting one in every ten newborns (9·9%),^(1)^ whereas the rate for Brazil is 9.2%.^(2)^ Infectious and noninfectious factors, personal and socioeconomic background and habits are consistently associated with preterm delivery (PTD).^(3)^

Inflammation, infection, uterine distention, decidual hemorrhage, decidual aging, disruption of maternal-fetal tolerance, reduction of progesterone, activation of the maternal-fetal hypothalamus-pituitary-adrenal axis, and gene interaction activate preterm birth.^(4)^

Biomarkers can be used to identify specific interventions early when physiological system malleability increases the likelihood of success. However, identifying reliable biomarkers, particularly in the perinatal period, including preterm infants, has been challenging.

We define a biomarker as a biological tool that can be quantified, measured, or evaluated to indicate differences in response to adverse experiences, and may aid in screening, diagnosis, prognosis, or biological response measurement. It should also have a high *positive likelihood ratio* (+ LR), increasing the probability that women are actually at risk of PTD, and a low negative likelihood ratio (- LR), to confidently rule out the disorder. A total of 42 biomarkers were identified as being associated with PTD.^(5)^

Cytokines are a diverse group of soluble non-antibody proteins that mediate inflammation and many other processes.^(6)^ These proteins exhibit pleiotropy and functional redundancy, up and down-regulating one another to result in complex networks involved in the establishment and maintenance of pregnancy^(7)^and complications such as miscarriage,^(8)^ preeclampsia^(9)^ and spontaneous PTD.^(10)^

A long-standing paradigm classifies cytokines based on their T-cell lineage as Th1 or Th2. The balance between these two types has been considered a measure of the immune milieu. Normal pregnancy has been characterized as a Th2-dominant state,^(11)^ although the time course of the expected shift to Th2 dominance in peripheral blood is not clear.^(12)^ A third T-cell lineage, named ‘Th17’ and its main product, interleukin (IL)-17, has been recently characterized.^(13)^ IL-17 is a potent pro-inflammatory cytokine that promotes the expansion and recruitment of neutrophils.^(14)^

Most studies that have evaluated cytokines as markers of PTD have not focused on T-cell lineage, and have instead applied the ‘pro-inflammatory’ label to include various Th-1 and Th-2 cytokines, chemokines and growth factors.^(15)^

Predictive biomarkers from maternal circulation would be valuable because blood is already routinely drawn several times during prenatal care, but studies evaluating these are sparse and less consistent. Generally, maternal mid-pregnancy biomarkers have been more strongly associated with earlier PTD.

Placental insufficiency is a common human pregnancy complication, leading to great obstetric syndromes: preeclampsia, fetal growth restriction, and preterm labor.

The objective of this study was to assess the relationship between placental insufficiency and a panel of mid-pregnancy biomarkers measured in maternal plasma, according to a Th1, Th2 and Th17 lineage framework, with the risk of PTD.

## Methods

This was a case-control study nested into the BRISA birth cohort study “Etiological factors of preterm birth and consequences of perinatal factors for infant health: birth cohort in two Brazilian cities”.^(16)^ Of 1370 mother-infant-placenta pairs in the Ribeirao Preto Medical School in Ribeirao Preto, Sao Paulo, Brazil in 2010, 82 were selected for detailed assessment of biological specimens using a sampling scheme designed to optimize available resources and maximize statistical power. Inclusion criteria were living in Ribeirão Preto, and giving informed consent to participate in the study. Exclusion criteria were multiple fetuses, congenital anomalies or chromosome syndromes, and loss of follow-up. Mothers with medical conditions (such as diabetes, smoking, alcohol or drug dependence, preeclampsia, or hypertension) were not excluded since the aim of the study was to describe community-based data rather than those of a ‘healthy’ population. A standardized questionnaire was used to record maternal characteristics. Gestational age was estimated according to the first day of the last menstrual cycle and modified if it disagreed by >2 weeks with ultrasound conducted before 25 weeks. Preterm (Yes/No) was defined in the total population as <37^0^ weeks of gestation, according to WHO.

At 20^0^ to 25^6^ weeks of gestation, women provided non-fasting venous blood samples. These were collected into EDTA or clot tubes, spun in cooled centrifuges and immediately frozen. Assays of maternal serum were performed using a multiplex immunoassay based on Luminex flowmetric xMAP technology.^(17)^ Luminex x MAP (multiple analyte profiling) was developed for the study of inflammatory reactions in newborns^(17)^ and has been used to measure circulating cytokine levels in pregnant women.^(18)^ For the present analysis, we selected a group of cytokines that could be classified as Th1, Th2 or Th17 to study its relation with PTD.^(19)^ The Th1-related cytokines selected were IFN-γ, IL-1β, IL-2, IL-6, IL-8, IL-12P40, TNF–α and TNF–β. The Th2-related cytokines selected were IL-4, IL-5, IL-10 and IL-13. To represent Th17, we selected IL-17A. Other biomarkers were GCSF, MCP1, MIP1A, MIP1B, RANTES, VEGF, EGF, FGF, and GMSF (Chart 1). This is a conceptual but probably over-simplified classification scheme given the complex cross-talk among cytokines.

**Chart 1:** Biomarkers


INF-γ Interferon-gamma, IL-2 Interleukin 2, IL-6 Interleukin 6, IL-8 Interleukin 8, IL-12P40 IL12 p40, IL-1B cytokine encoded by the IL1B gene, TNF Tumor necrosis factors α and β, IL-4 Interleukin 4, IL-5 Interleukin 5, IL-10 Interleukin 10, IL-13 Interleukin 13, IL-17A Interleukin 17A, GCSF Granulocyte colony-stimulating factor, MCP1 Monocyte chemoattractant protein 1, MIP1 Macrophage Inflammatory proteins 1 α and β, RANTES Regulated on activation normal T cell expressed and secreted, VEFG Vascular endothelial growth factor, EGF Epidermal Growth Factor, FGF Fibroblast growth factor, GMSF Granulocyte-macrophage colony-stimulating factor

Placentas were weighed in grams (g) on an electronic scale after cutting the cord and membranes by the Department of Pathology according to standardized procedures;^(20)^ measurements were obtained blindly from pregnancy and delivery data. The ratio between birth weight (BW) and placental weight (PW) in grams (BW/PW) was used as a proxy for *placental efficiency*. The values were distributed according to gestational age (GA), and BW/PW ratio values below the lower quartile were considered indicators of low placental efficiency.^(21)^

**Figure 1:**
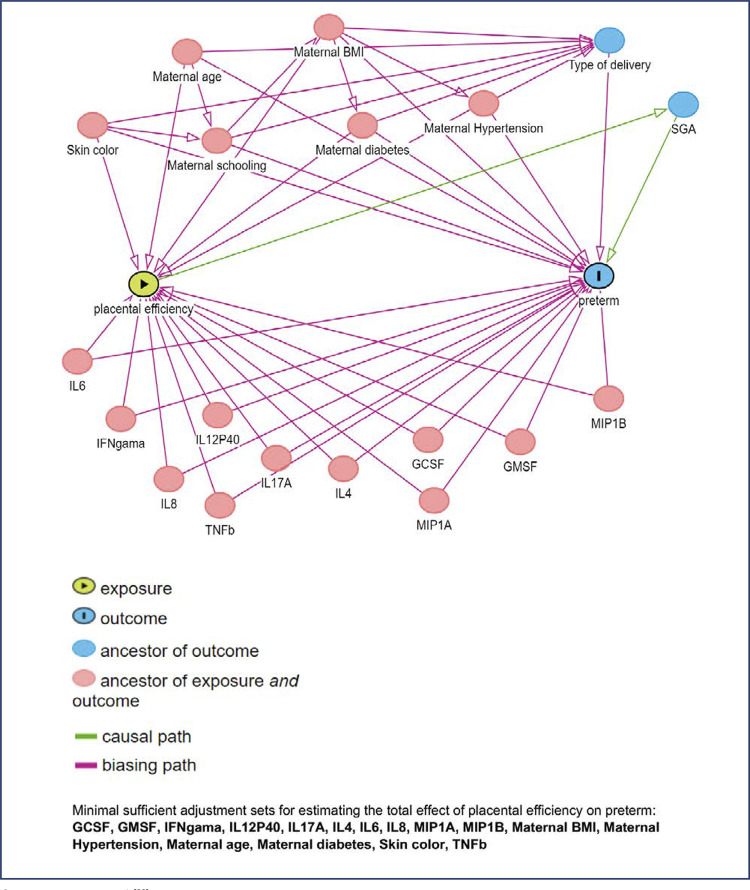
Directed Acyclic Graph

Source: Textor et al.^(22)^

Mother demographics and obstetric history, newborn characteristics, biomarkers and placental efficiency are reported descriptively as mean and standard deviation (SD) or frequencies and proportions. Spearman correlation coefficients were calculated for each pair of 23 biomarkers. Univariate analyses for the association between preterm delivery and biomarkers were performed using logistic regression models, and odds ratios (OR) and 95% confidence intervals were calculated. Adjusted analyses were further evaluated for preterm birth and covariates of interest, which were selected a priori based on a Directed Acyclic Graph (DAG) generated with DAGitty version 2.3 (Chart 2) (Figure 1).^(22)^ Confounders included mother’s education, skin color, BMI, diabetes, hypertension and placental efficiency.

**Chart 2:** Correlation matrix

		Th1								Th2				Th17	Other biomarkers								
		IFNgama	IL2	IL6	IL8	IL12040	IL1B	TNFa	TNFbeta	IL4	IL5	IL10	IL13	IL17A	GCSF	MCP1	MIP1A	MIP1B	RANTES	VEGF	EGF	FGF	GMSF
h1: Pro-inflammatory	IFNgama	1	0,08	0,23*	0,78*	0,15	0,21	0,51*	0,03	0,13	0,28*	0,31*	-0,03	0,91*	0,53*	0,14	0,26*	0,58*	0,29*	0,48*	0,18	0,47*	0,52*
	IL2	0,08	1	0,75*	0,28*	0,70*	0,73*	0,36*	0,69*	0,75*	0,55*	0,40*	0,79*	0,11	0,42*	0,13	0,44*	0,54*	-0,25*	0,08	-0,08	0,55*	0,49
	IL6	0,23*	0,75*	1	0,37*	0,77*	0,70*	0,35*	0,65*	0,79*	0,44*	0,56*	0,73*	0,24*	0,47*	0,07	0,42*	0,59*	-0,07	0,1	-0,22*	0,55*	0,55*
	IL8	0,78*	0,28*	0,37*	1	0,33*	0,36*	0,60*	0,29*	0,36*	0,40*	0,54*	0,21	0,81*	0,65*	0,29*	0,36*	0,74*	0,23*	0,22*	0,11	0,63*	0,67*
	IL12P40	0,15	0,70*	0,77*	0,33*	1	0,70*	0,26*	0,80*	0,92*	0,35*	0,56*	0,83*	0,15	0,58*	0,07	0,31*	0,54*	0,01	-0,55	-0,22*	0,53*	0,72*
	IL1B	0,21	0,73*	0,70*	0,36*	0,70*	1	0,34*	0,65*	0,74*	0,56*	0,56*	0,72*	0,25*	0,51*	0,16	0,35*	0,61*	-0,09	0,08	-0,02	0,58*	0,57*
	TNFa	0,51*	0,36*	0,35*	0,60*	0,26*	0,34*	1	0,17	0,21	0,61*	0,44*	0,24*	0,49*	0,72*	0,38*	0,57*	0,74*	-0,02	0,30*	0,36*	0,73*	0,46*
	TNFbeta	0,03	0,69*	0,65*	0,29*	0,80*	0,65*	0,17	1	0,85*	0,32*	0,47*	0,83*	0,04	0,48*	0,09	0,30*	0,47*	-0,02	-0,05	-0,22*	0,45*	0,57*
Th2: Anti-inflammatory	IL4	0,13	0,75*	0,79*	0,36*	0,92*	0,74*	0,21	0,85*	1	0,37*	0,59*	0,86*	0,13	0,50*	0,09	0,25*	0,51*	-0,01	-0,06	0,24*	0,50*	0,72*
	IL5	0,28*	0,55*	0,44*	0,40*	0,35*	0,56*	0,61*	0,32*	0,37*	1	0,42*	0,49*	0,33*	0,54*	0,23*	0,47*	0,57*	-0,28*	0,22*	0,32*	0,64*	0,34*
	IL10	0,31*	0,40*	0,56*	0,54*	0,56*	0,56*	0,44*	0,47*	0,59*	0,42*	1	0,51*	0,31*	0,55*	0,41*	0,23*	0,59*	0,07	-0,01	-0,07	0,61*	0,58*
	IL13	-0,03	0,79*	0,73*	0,21	0,83*	0,72*	0,24*	0,83*	0,86*	0,49*	0,51*	1	-0,03	0,43*	0,11	0,40*	0,46*	-0,13	-0,12	-0,1	0,52*	0,52*
Th17: Pro-inflammatory	IL17A	0,91*	0,11	0,24*	0,81*	0,15	0,25*	0,49*	0,04	0,13	0,33*	0,31*	-0,03	1	0,48*	0,16	0,25*	0,58*	0,27*	0,52*	0,2	0,44*	0,50*
Other biomarkers	GCSF	0,53*	0,42*	0,47*	0,65*	0,58*	0,51*	0,72*	0,48*	0,50*	0,54*	0,55*	0,43*	0,48*	1	0,35*	0,51*	0,73*	0,1	0,13	0,12	0,79*	0,73*
	MCP1	0,14	0,13	0,07	0,29*	0,07	0,16	0,38*	0,09	0,09	0,23*	0,41*	0,11	0,16	0,35*	1	0,1	0,25*	-0,08	0,07	0,18	0,40*	0,2
	MIP1A	0,26*	0,44*	0,42*	0,36*	0,31*	0,35*	0,57*	0,30*	0,25*	0,47*	0,23*	0,40*	0,25*	0,51*	0,1	1	0,53*	-0,11	0,28*	0,24*	0,55*	0,29*
	MIP1B	0,58*	0,54*	0,59*	0,74*	0,54*	0,61*	0,74*	0,47*	0,51*	0,57*	0,59*	0,46*	0,58*	0,73*	0,25*	0,53*	1	-0	0,23*	0,14	0,79*	0,69*
	RANTES	0,29*	-0,25*	-0,07	0,23*	0,01	-0,09	-0,02	-0,02	-0,01	-0,28*	0,07	-0,13	0,27*	0,1	-0,1	-0,11	-0,004	1	-0,0005	0,03	-0,06	0,23*
	VEGF	0,48*	0,08	0,1	0,22*	-0,55	0,08	0,30*	-0,05	-0,06	0,22*	-0,01	-0,12	0,52*	0,13	0,07	0,28*	0,23*	-0	1	0,2	0,16	0,08
	EGF	0,18	-0,08	-0,22*	0,11	-0,22*	-0,02	0,36*	-0,22*	0,24*	0,32*	-0,07	-0,1	0,2	0,12	0,18	0,24*	0,14	0,03	0,2	1	0,16	0,01
	FGF	0,47*	0,55*	0,55*	0,63*	0,53*	0,58*	0,73*	0,45*	0,50*	0,64*	0,61*	0,52*	0,44*	0,79*	0,40*	0,55*	0,79*	-0,06	0,16	0,16	1	0,68*
	GMSF	0,52*	0,49	0,55*	0,67*	0,72*	0,57*	0,46*	0,57*	0,72*	0,34*	0,58*	0,52*	0,50*	0,73*	0,2	0,29*	0,69*	0,23*	0,08	0,01	0,68*	1

*means significantly at p<0.05

A receiver operating characteristic (ROC) curve was constructed for each biomarker and the corresponding area under the curve (AUC, 95 % confidence interval [CI]) was calculated. Also, sensitivity, specificity, positive and negative likelihood ratios (+LR, -LR) were calculated. We agree that when a test is used either for the purpose of screening or to exclude a diagnostic possibility, a cut-off value with a high sensitivity may be selected. Finally, the Youden index, a summary measure of the ROC curve that measures the effectiveness of a diagnostic marker and enables the selection of an optimal threshold value (*cutoff point*) was calculated; its value can be from -1(useless) to 1 (perfect). Interaction models to evaluate the *effect modification* were performed; all models included a product term: placental efficiency * interleukins. All analyses were performed using Stata 14.01 (College Station, Texas, USA) and SAS/STAT1 (SAS Institute Inc., Cary, NC, 2010) for all statistical calculations, with the level of significance set at 0.05. We followed the STROBE recommendations for reporting observational studies (https://www.strobe-statement.org/).

The project was approved by the Research Ethics Committee of the Ribeirão Preto Medical School, at the University of São Paulo, Brazil (process No. 11157/2008).

## Results

The mean maternal age was 27.9 (SD 6.06) years. Almost half of the mothers were non-white, overweight or obese, attended school was higher for preterm mothers (p = 0,045), 36,6% (n=30) were primiparous, 30,5% (n=25) had hypertension, and diabetes was higher for term mothers (p = 0,024) (Table 1).

**Table 1:** Maternal characteristics

Characteristics	Total (n=82) n(%)	Term (control) (n= 37) n(%)	Preterm (n= 45) n(%)	p-value*
Skin color				0.96
Non-white	43(53.0)	19(52.78)	24(53.33)	
White	38(46.91)	17(47.22)	21(46.67)	
Age (years)				0.408
20-34	64(78.05)	31(83.78)	33(73.33)	
<20	5(6.10)	1(2.70)	4(8.89)	
>35	13(15.85)	5(13.51)	8(17.78)	
IMC (kg/m^2^)				0.116
Underweight (<18.5)	3(4.23)	2(6.67)	1(2.44)	
Normal (> 18.5 - < 25)	33(46.48)	11(36.67)	22(53.66)	
Overweight (> 25 - <30)	15(21.13)	10(33.33)	5(12.20)	
Obesity (> 30)	20(28.17)	7(23.33)	13(31.71)	
Education (y)				0.045
> 12	6(7.59)	5(14.29)	1(2.27)	
<12	73(92.41)	30(85.71)	43(97.73)	
Parity (n)				0.344
1	30(36.59)	12(32.43)	18(40.00)	
2 - 3	42(51.22)	22(59.46)	20(44.44)	
> 4	10(12.20)	3(8.11)	7(15.56)	
Diabetes	12(14.63)	9(24.32)	3(6.67)	0.024
Hypertension	25(30.49)	12(32.43)	13(28.89)	0.729
Anemia	10(12.35)	3(8.33)	7(15.56)	0.326
Urinary infection	29(35.37)	12(32.43)	17(37.78)	0.614
Delivery				0.072
Vaginal	42(51.22)	23(62.16)	19(42.22)	
Cesarean	40(48.78)	14(37.84)	26(57.78)	

*chi-square test. Data in bold means significantly at p<0.05

The mean gestational age was 250 (SD 30) days. As expected, the average birth weight, length and BMI were higher for term newborns (all p< 0,001); 14,6% were *small for gestational* (SGA), higher for term newborns (p=0,029), 4,8% *large for gestational age* (LGA), and 13,4% *stunted*, where neither showed *wasting* (Table 2), suggesting a high-risk pregnancy.

**Table 2:** Newborn characteristics

Characteristics	Total (n=82) n(%)	Term (n= 37) n(%)	Preterm (n= 45) n(%)	p-value*
Male	38 (46,34)	16 (43,24)	22 (48,89)	0,610
Gestational age (days)	250 (30)	275 (9)	230 (26)	<0,0001
Weight (g)	2580 (967)	3285 (465)	2001 (886)	<0,0001
Length (cm)	45,8 (5)	48,7 (2,3)	42,7 (5,1)	<0,0001
BMI (kg/m^2^)	12,8 (1,9)	13,8 (1,3)	11,8 (2,0)	0,0001
Phenotypes according to IG-21^**^				
SGA (weight <3^rd^ percentile)	7 (8,5)	1 (2,7)	6 (13,3)	0,086
SGA (weight < 10^th^ percentile)	12 (14,6)	2 (5,4)	10 (22,2)	0,029
LGA (weight > 90^th^ percentile)	4 (4,8)	1 (2,7)	3 (6,6)	0,394
Stunted (length < 3^rd^ percentile)	11 (13,4)	3 (8,1)	8 (17,7)	0,340
Wasted (BMI < 3^rd^ percentile)	0	0	0	-

Data expressed as mean (Standard Deviation) or count (percentage); *Student T test or chi-square test when corresponding; Data in bold means significantly at p<0.05; **Reference 23; SGA small for gestational age, LGA large for gestational age, BMI body mass index

Placental efficiency was higher for term newborns (p<0,001) and increased from 22-28 weeks to 37-42 weeks (p<0,001) (Table 3).

**Table 3:** Placental efficiency

	n	Mean	Standard Deviation	p-value^*^
Total	81	4,81	1,28	
Term	37	5,38	1,04	0,001
Preterm	44	4,33	1,28	
Gestational age (w)				0,001
22-28	9	2,78	1,16	
29-32	8	4,04	0,97	
33-36	27	4,39	0,92	
37- 42	36	5,39	1,05	

*Student T test; Data in bold means significantly at p<0.05

### Biomarkers (Chart 3)

**Chart 3:** Comparison of the mean levels and standard error of 22 biomarkers between women with preterm birth (PTB Group) and women who delivered at term (Control Group)

Th1: pro-inflammatory				
Biomarker	Total n = 82 Mean (SE)	Preterm n = 37 Mean (SE)	Term n = 45 Mean (SE)	p-value*
IFNgama	89.70(14.86)	110.37(27.70)	72.70(14.51)	0.20
IL-2	10.82(2.55)	14.28(5.51)	7.98(1.00)	0.22
IL-6	23.02(2.29)	23.81(3.03)	22.36(3.39)	0.75
IL-8	65.05(9.58)	93.68(17.40)	41.50(8.73)	0.003
IL-12P40	52.49(4.97)	65.51(8.07)	41.78(5.79)	0.01
IL-1B	6.44(0.51)	6.96(0.84)	6.01(0.62)	0.36
TNFa	16.14(1.12)	16.82(1.49)	15.59(1.66)	0.59
TNFbeta	34.62(6.63)	46.29(13.67)	25.03(4.12)	0.11
Th2: anti-inflammatory				
IL-4	70.36(11.42)	98.15(19.37)	47.51(12.63)	0.02
IL-5	5.23(0.79)	4.45(0.52)	5.86(1.37)	0.37
IL-10	12.96(1.24)	13.07(1.16)	12.87(2.07)	0.93
IL-13	17.51(2.58)	23.06(5.13)	12.96(1.88)	0.02
Th17: pro-inflammatory				
IL-17A	47.80(8.67)	62.39(16.26)	35.80(8.19)	0.12
Other biomarkers				
GCSF	189.72(12.20)	220.13(21.05)	164.72(13.04)	0.02
MCP1	483.69(26.48)	477.74(32.11)	488.58(40.71)	0.84
MIP1A	28.00(9.75)	42.48(21.43)	16.09(1.76)	0.18
MIP1B	109.20(11.08)	136.82(18.74)	86.50(12.23)	0.03
RANTES	11973.63(682)	12779(1059)	11310(885)	0.28
VEGF	367.50(34.08)	362.57(55.94)	371.55(42.32)	0.89
EGF	186.02(14.32)	178.09(14.06)	196.66(23.44)	0.41
FGF	166.36(10.16)	187.14(16.93)	149.28(11.77)	0.06
GMSF	81.61(9.72)	106.94(17.00)	60.78(10.04)	0.01

All concentrations are in pg/mL except for RANTES in ng/mL; Data expressed as mean (Standard Error. SE); *Student T-test. Data in bold means significantly at p<0.05; Biomarkers: IFNgamma Interferon-gamma (INF-γ). IL-2 Interleukin 2. IL-6 Interleukin 6. IL-8 Interleukin 8. IL-12P40 IL12 p40. IL-1B cytokine encoded by the IL1B gene. TNF Tumor necrosis factors α and β. IL-4 Interleukin 4. IL-5 Interleukin 5. IL-10 Interleukin 10. IL-13 Interleukin 13. IL-17A Interleukin 17A. GCSF Granulocyte colony-stimulating factor. MCP1 Monocyte chemoattractant protein 1. MIP1 Macrophage Inflammatory proteins 1 α and β. RANTES Regulated on activation. normal T cell expressed and secreted. VEFG Vascular endothelial growth factor. EGF Epidermal Growth Factor. FGF Fibroblast growth factor. GMSF Granulocyte-macrophage colony-stimulating

Th1: all pro-inflammatory biomarkers values were higher for preterm than the control group, but only IL-8 and IL-12P40 were statistically significant.

Th2: of this lineage four out of five present higher values for preterm, two statistically significant.

Th17 showed twice the value in preterm than the control group, in de border of significance.

Finally, in the other biomarkers group six out of nine present higher values for preterm, three statistically significant.

In summary, in the present study, 18/22 (81%, 95% CI 61 – 92) preterm biomarker values were higher than the control group, being 7/18 (38%) statistically significant. Th1 cytokines were highly correlated, while the weakest correlation was observed in the *other biomarkers*, but these correlations were not necessarily stronger within classification groups than between them. Risk of preterm birth for selected clinical characteristics, placenta insufficiency and biomarkers (Table 4).

**Table 4:** Risk of preterm birth for selected clinical characteristics, placenta insufficiency and biomarkers

Variables	Unadjusted OR	95% CI	p-value	Adjusted OR	95% CI	p-value
Clinical characteristics						
Education <12 y	7.16	0.79 – 64.5	0.079	9.40	1.04 – 93.9	0.046
Diabetes	0.22	0.05 – 0.89	0.034	0.22	0.04 – 1.11	0.068
Hypertension	0.84	0.33 – 2.17	0.729	0.88	0.25 – 3.07	0.847
Cesarean delivery	2.24	0.92 – 5.47	0.074	3.07	0.81 – 11.5	0.096
Placental insufficiency	0.87	0.31 – 2.42	0.790	0.56	0.16 – 1.93	0.363
Proinflammatory cytokines Th1						
IL-2	0.97	0.92 – 1.03	0.401	0.97	0.89 – 1.06	0.569
IFNgama	0.99	0.99 – 1.00	0.224	0.99	0.99 – 1.00	0.571
IL-6	0.99	0.97 – 1.01	0.753	0.98	0.96 – 1.01	0.328
IL-8	0.99	0.98 – 0.99	0.012	0.99	0.98 – 0.99	0.027
IL12P40	0.98	0.97 – 0.99	0.021	0.98	0.97 – 0.99	0.027
IL-1β	0.95	0.86 – 1.05	0.360	0.90	0.79 – 1.04	0.186
TNFα	0.98	0.94 – 1.03	0.588	0.98	0.94 - 1.03	0.661
TNFβ	0.98	0.97 – 1.00	0.166	0.97	0.94 – 0.99	0.049
Anti-inflammatory cytokines Th2						
IL-4	0.99	0.98 – 0.99	0.031	0.99	0.98 – 0.99	0.017
IL-5	1.03	0.96 – 1.10	0.390	1.02	0.93 – 1.11	0.621
IL-10	0.99	0.96 – 1.03	0.936	0.99	0.94 – 1.03	0.729
IL-13	0.97	0.94 – 1.00	0.067	0.96	0.92 – 0.99	0.047
Proinflammatory cytokines Th17						
IL17A	0.99	0.98 – 1.00	0.146	0.99	0.98 -1.01	0.193
Other biomarkers						
GCSF	0.99	0.98 – 0.99	0.029	0.99	0.99 – 0.99	0.039
GMSF	0.99	0.98 – 0.99	0.024	0.36	0.16 – 0.82	0.015
MCP1	1.00	0.99 – 1.00	0.838	1.00	0.99 – 1.00	0.921
MIP1A	0.97	0.93 – 1.00	0.103	0.97	0.92 – 1.03	0.437
MIP1B	0.99	0.98 – 0.99	0.027	0.99	0.98 – 0.99	0.027
RANTES	0.99	0.99 – 1.00	0.285	0.99	0.99 – 1.00	0.359
VEGF	1.00	0.99 – 1.00	0.895	1.00	0.99 – 1.00	0.668
EGF	1.00	0.99 – 1.00	0.414	1.00	0.99 – 1.00	0.895
FGF	0.99	0.99 – 1.00	0.066	0.99	0.98 – 0.99	0.014

Adjusted for education, skin color, BMI, diabetes, hypertension and placental insufficiency. Data in bold means significantly at p<0.05. *Biomarkers*: IL-2 Interleukin 2, IFNgamma Interferon-gamma (INF-γ), IL-6 Interleukin 6, IL-8 Interleukin 8, IL-12P40 IL12 p40, IL-1β cytokine encoded by the IL1β gene, TNF Tumor necrosis factors α and β, IL-4 Interleukin 4, IL-5 Interleukin 5, IL-10 Interleukin 10, IL-13 Interleukin 13, IL-17A Interleukin 17A, GCSF Granulocyte colony-stimulating factor, GMSF Granulocyte-macrophage colony-stimulating factor, MCP1 Monocyte chemoattractant protein 1, MIP1 Macrophage Inflammatory proteins 1 α and β, RANTES Regulated on activation, normal T cell expressed and secreted, VEFG Vascular endothelial growth factor, EGF Epidermal Growth Factor, FGF Fibroblast growth factor

Less education was associated with a ten times higher risk of PTD (p = 0.046), whereas no appreciable difference in the association with PTD was observed for *placental insufficiency*. Biomarkers showed negligible adjusted OR (aOR), ranging from 0.90 to 1.02; *proinflammatory cytokines* IL-8, IL12P40 and TNFβ, *anti-inflammatory cytokines* IL-4 and IL-13, and GCSF, GMSF, MIP1B and FGF were significantly associated with lower risk of preterm birth. S*ensitivity analysis* was performed to assess the risk of preterm birth associated with different neonatal *phenotypes*. In adjusted models SGA *<*3^rd^ percentile according to Intergrowth-21^st^,IL-4 showed an aOR of 0.91 (95% CI 0.83-0.99). For SGA<10^th^percentile the risk for IFNgama was 1.04 (1.00-1.08) and for IL-17A 0.83 (0.72-0.97), all statistically significant differences. LGA age was not associated with PTD. Finally, in the *stunted* model (height <3^rd^ percentile), MIP1B presented an aOR of 0.90 (0.83 to 0.98). To obtain standardized measures, we divided biomarker values by their standard deviation within the controls. The resulting standardized values were approximately normally distributed, but no differences were observed between term and preterm newborns (data not shown). Thirteen models explored the *effect modification* between placental efficiency and interleukins, but only TNFa showed a significant aOR (0.81, 95% CI 0.67- 0.97). However, the results were nonsignificant for the other biomarkers. Proinflammatory IL-8 and IL12P40, anti-inflammatory IL-4 and GSF, MIP1B and GMCSF showed higher and statistically significant AUC ranging from 0.62 to 0.68. On the other hand, proinflammatory IL-6, IL-8, IL-1β and TNFβ, anti-inflammatory IL-4 and IL-13, and GCSF, MIP1A, VEGF, EGF and FGF2 presented higher *sensitivity* ranging from 75.56% to 91.11%. Youden index ranged between 0.115 and 0.371, while the higher +LR was 3.29 (GMCSF) and the lower -LR was 0,25 (GCSF) (Chart 4).

**Chart 4:** Prognostic accuracy of selected biomarkers

Biomarker	AUC	95% CI	p-level*	Youden index	Cut off	Sensitivity	Specificity	+LR	-LR
Th1: pro-inflammatory									
IFNgama	0,58	0,467 to 0,689	0,211	0,212	≤57,69	75,56	51,35	1,55	0,48
IL2	0,57	0,452 to 0,675	0,305	0,198	≤8,64	71,11	48,65	1,38	0,59
IL6	0,57	0,453 to 0,676	0,295	0,169	≤39,17	84,44	32,43	1,25	0,48
IL8	0,64	0,526 to 0,743	0,028	0,299	≤67,13	86,67	43,24	1,53	0,31
IL12P40	0,65	0,534 to 0,750	0,018	0,325	≤34,74	62,22	70,27	2,09	0,54
IL1B	0,57	0,453 to 0,676	0,300	0,174	≤7,54	82,22	35,14	1,27	0,51
TNFa	0,57	0,453 to 0,676	0,301	0,160	≤12,09	51,11	64,86	1,45	0,75
TNFbeta	0,61	0,496 to 0,716	0,083	0,218	≤36,69	86,67	35,14	1,34	0,38
Th2: anti-inflammatory									
IL4	0,63	0,517 to 0,734	0,036	0,218	≤166,74	86,67	35,14	1,34	0,38
IL5	0,52	0,403 to 0,628	0,813	0,148	≤3,95	68,89	45,95	1,27	0,68
IL10	0,60	0,482 to 0,703	0,141	0,315	≤9,88	66,67	64,86	1,9	0,51
IL13	0,59	0,479 to 0,700	0,152	0,272	≤21,1	86,67	40,54	1,46	0,33
Th17: pro-inflammatory									
IL17A	0,61	0,491 to 0,711	0,100	0,247	≤25,8	73,33	51,35	1,29	0,62
Other biomarkers									
GCSF	0,62	0,509 to 0,727	0,052	0,263	≤294,43	91,11	35,14	1,40	0,25
MCP1	0,51	0,401 to 0,626	0,827	0,115	≤409,68	46,67	64,86	1,33	0,82
MIP1A	0,61	0,493 to 0,713	0,093	0,177	≤30,26	93,33	24,32	1,23	0,27
MIP1B	0,64	0,525 to 0,742	0,024	0,229	≤67,09	68,89	54,05	1,5	0,58
RANTES	0,57	0,458 to 0,681	0,265	0,247	≤12459	73,33	51,35	1,51	1,51
VEGF	0,58	0,467 to 0,689	0,220	0,237	>221,52	77,78	45,95	1,44	0,48
EGF	0,51	0,396 to 0,621	0,887	0,195	≤319,34	77,78	2,7	0,80	8,22
FGF2	0,60	0,488 to 0,708	0,112	0,201	≤202,63	82,22	37,84	1,32	0,47
GMCSF	0,68	0,567 to 0,778	0,003	0,371	0,3712	53,33	83,78	3,29	0,56

*p level <.05 in red color; AUC - area under the ROC curve; CI - confidence interval

## Discussion

Higher second-trimester serum levels of IL-8 IL-12p40, IL-4, IL-13, GCSF, MIP1B and GMSF in asymptomatic pregnant women were statistically associated with PTB. Classical maternal risk factors were associated with PTB, whereas the neonatal phenotypes were more frequent in the preterm group. There is increasing evidence that placental efficiency changes over time in any given population and is likely to be influenced by the nutritional environment. In a previous study, the BW/PW ratio below the lower tercile was associated with an increased risk of pre-eclampsia, induced labor, cesarean section, and PTD (p<0.001).^(23-25)^The *causes of placental insufficiency* are unknown, but there is evidence of association with placental inflammation, hypoxia, and subsequent oxidative and/or nitrative stress.^(26)^ However, the mechanistic pathways underlying placental dysfunction have not been fully elucidated. Chronic inflammatory lesions of the placenta are characterized by the infiltration of the organ by lymphocytes, plasma cells, macrophages, and maternal T cells (CD8 + cytotoxic T cells), and are driven by the production of T-cell chemokines in the affected lesions. It has been reported in association with preterm and term fetal growth restriction, preeclampsia, fetal death, and preterm labor. Chronic chorioamnionitis is the most common lesion in late spontaneous preterm birth and is characterized by the infiltration of maternal CD8 + T cells into the chorioamniotic membranes.^(27)^

In the present study, most of the included biomarkers were cytokines, chemokines, growth and angiogenic-stimulating factors, and adhesion molecules known to be involved with PTD. We found that 81% of preterm biomarker values were higher than in the term control group, and IFN-γ, GCSF, MIP1B, RANTES, and FGF showed the higher concentrations compared to women who delivered at term. It has long been established that a *bias* from the T1 helper cytokine profile towards the T2 helper profile contributes to successful pregnancy maintenance. Over the last few decades, there has been an increased awareness of the role of this balance in PTD. However, not all studies support this hypothesis. Shimaoka et al. reported a reduction in IL-4 during pregnancy,^(28)^ while Matthiesen et al.^(29)^ presented data suggesting an increase in both IL-4 and IFN-γ secreting cells in pregnancy compared with nonpregnant controls. Such discrepancies may be due to the characterization of cytokine profiles in either isolated cell populations or whole blood.

It is important to note that, although the Th1 and Th2 responses can be seen as discrete responses, there is considerable crosstalk and overlap between the functions of the T helper cells. The *hypothesis* of Th2 predominance and downregulation of the Th1 response originated from Wegmann et al.^(30)^ was reinforced by evidence from both murine studies and the clinical course of Th2 and Th1-based conditions in pregnancy.

There is substantial evidence that the *Th1 cytokines* play a role in the initiation of labor at term.^(11)^ In the context of pregnancy, interleukin 8 is thought to attract leukocytes to the gestational tissues and the cervix at the onset of term and preterm labor. IL-8 mRNA expression has been reported to be increased more than 50-fold in preterm labor.^(31)^ In the present study, IL-8 in premature doubled the value of controls. IL-12p40 is produced primarily by activated inflammatory cells including macrophages, neutrophils, microglia and dendritic cells.^(32)^ IL-4 is considered to be an anti-inflammatory cytokine and is commonly associated with strong antibody responses, for example, stimulating IgE and IgG1 antibody production.^(28)^ Biomarkers of *inflammation*, such as levels of amniotic fluid IL-6 show improved sensitivities compared to amniotic fluid culture and may positively correlate with histologic evidence of chorioamnionitis.^(33)^ Finally, a study comparing women in active preterm labor and no labor showed no difference in median cytokine IL-13 production.^(34)^

Taken together, these results suggest that, rather than a decrease in the Th2 response, preterm labor most likely represents an activation of the Th1 response. In adjusted analysis maternal education differed significantly between the case and control groups, whereas biomarkers showed negligible aOR. These findings might be related to the small sample size, the choice of the control group, and the fact that a convenience cohort was studied. In a similar study from Brazil, among the 41 cytokines analyzed, only growth-related oncogene (GRO) was a risk factor for spontaneous PTB.^(35)^

Importantly, eleven (50%) biomarkers presented a high *sensitivity* ranging from 75.56% to 91.11%, also Youden index showed a weak diagnostic capacity. A high-risk cohort study concluded that five cytokines sampled from mid-pregnancy maternal serum in low-risk women offered little useful predictive value of PTB.^(36)^ Positive LR values increased the probability of PTD by a small amount; on the other hand, negative LR values resulted in a weak probability of discarding PTD.

There are not many articles in pregnancy literature about specific biomarkers and placenta jointly related to preterm birth. Although a biomarker may allow the use of a small sample size, *confounding factors*, particularly the use of drugs, can negate the value of biomarkers. In order to analyze all cytokines using a common scale and reduce the impact of intraassay variability, biomarker values were standardized.

*Limitations:* Normal cut-off values for the cytokines are not available in the literature for direct comparison with the outcome (prematurity). The small sampling can explain the lack of association, mainly because of an underpowered study, with a wide CI.

Several biomarkers in maternal and fetal compartments have been mechanistically linked to PTD, but none of them are reliable predictors of pregnancy outcome.^(19)^Additional follow-up monitoring is needed to determine whether the association found in this study persists into early childhood and beyond and may help predict future long-term disorders, such as failure to thrive, neurodevelopmental disabilities, metabolic syndrome, and cardiovascular diseases.

## Conclusion

Higher second-trimester serum levels of IL-8, IL-12p40, IL-4, IL-13, GCSF, MIP1B and GMSF in asymptomatic pregnant women are associated with PTB. This finding suggests an activation of maternal inflammatory response.

## References

[1] Lawn J, Ohuma E, Bradley E, Idueta LS, Hazel E, Okwaraji YB (2023). Small babies, big risks: global estimates of prevalence and mortality for vulnerable newborns to accelerate change and improve counting. Lancet.

[2] Mendoza Tascón LA, Claros Benítez DI, Mendoza Tascón LI, Arias Guatibonza MD, Peñaranda Ospina CB (2016). Epidemiología de la prematuridad, sus determinantes y prevención del parto prematuro. Rev Chil Obstet Ginecol.

[3] Frey HA, Klevanoff MA (2016). The epidemiology, etiology, and costs of preterm birth. Semin Fetal Neonatal Med.

[4] Esplin MS (2014). Overview of spontaneous preterm birth: a complex and multifactorial phenotype. Clin Obstet Gynecol.

[5] Aronson JK, Ferner RE (2017). Biomarkers-a general review. Curr Protoc Pharmacol.

[6] Balkiwill F (2000). The cytokine network.

[7] Kharfi A, Giguere Y, Sapin V, Masse J, Dastugue B, Forest JC (2003). Trophoblastic remodeling in normal and preeclamptic pregnancies: implication of cytokines. Clin Biochem.

[8] Jasper MJ, Tremellen KP, Robertson SA (2007). Reduced expression of IL-6 and IL-1alpha mRNAs in secretory phase endometrium of women with recurrent miscarriage. J Reprod Immunol.

[9] Saito S, Shiozaki A, Nakashima A, Sakai M, Sasaki Y (2007). The role of the immune system in preeclampsia. Mol Aspects Med.

[10] Romero R, Espinoza J, Goncalves LF, Kusanovic JP, Friel LA, Nien JK (2006). Inflammation in preterm and term labor and delivery. Semin Fetal Neonatal Med.

[11] Wilczynski JR (2005). Th1/Th2 cytokines balance--yin and yang of reproductive immunology. Eur J Obstet Gynecol Reprod Biol.

[12] Aris A, Lambert F, Bessette P, Moutquin JM (2008). Maternal circulating interferon-gamma and interleukin-6 as biomarkers of Th1/Th2 immune status throughout pregnancy. J Obstet Gynaecol Res.

[13] Veldhoen M, Stockinger B (2006). TGFbeta1, a 'Jack of all trades': the link with pro-inflammatory IL-17-producing T cells. Trends Immunol.

[14] Cua DJ, Kastelein RA (2006). TGF-beta, a 'double agent' in the immune pathology war. Nat Immunol.

[15] Arntzen KJ, Kjollesdal AM, Halgunset J, Vatten L, Austgulen R (1998). TNF, IL-1, IL-6, IL-8 and soluble TNF receptors in relation to chorioamnionitis and premature labor. J Perinat Med.

[16] da Silva AA, Simões VM, Barbieri MA, Cardoso VC, Alves CM, Thomaz EB (2014). A protocol to identify non-classical risk factors for preterm births: the Brazilian Ribeirão Preto and São Luís prenatal cohort (BRISA). Reprod Health.

[17] Skogstrand K, Thorsen P, Norgaard-Pedersen B, Schendel DE, Sorensen LC, Hougaard DM (2005). Simultaneous measurement of 25 inflammatory markers and neurotrophins in neonatal dried blood spots by immunoassay with xMAP technology. Clin Chem.

[18] Vogel I, Goepfert AR, Thorsen P, Skogstrand K, Hougaard D, Curry A (2007). Early second-trimester inflammatory markers and short cervical length and the risk of recurrent preterm birth. J Reprod Immunol.

[19] Polettini J, Cobo T, Kacerovsky M, Vinturache A, Laudanski P, Peelen M (2016). Biomarkers of spontaneous preterm birth: a systematic review of studies using multiplex analysis. J Perinat Med.

[20] Benirschke K, Burton GJ, Baergen RN (2012). Pathology of the human placenta.

[21] Salafia CM, Yampolsky M (2009). Metabolic scaling law for fetus and placenta. Placenta.

[22] Textor J, Hardt J, Knüppel S (2011). DAGitty: a graphical tool for analyzing causal diagrams. Epidemiology.

[23] Villar J, Ismail LC, Victora CG, Ohuma E, Bertino E, Altman D (2014). International standards for newborn weight, length, and head circumference by gestational age and sex: the Newborn Cross-Sectional Study of the INTERGROWTH-21st Project. Lancet.

[24] Whitcomb BW, Schisterman EF, Klebanoff MA, Baumgarten M, Rhoton-Vlasak A, Luo X (2007). Circulating chemokine levels and miscarriage. Am J Epidemiol.

[25] Alwasel SH, Abotalib Z, Aljarallah JS, Osmond C, Alkharaz SM, Alhazza IM (2011). Secular increase in placental weight in Saudi Arabia. Placenta.

[26] Tong Y, Zhang S, Riddle S, Zhang L, Song R, Yue D (2021). Intrauterine hypoxia and epigenetic programming in lung development and disease. Biomedicines.

[27] Kim CJ, Romero R, Chaemsaithong P, Kim J (2015). Chronic inflammation of the placenta: definition, classification, pathogenesis, and clinical significance. Am J Obstet Gynecol.

[28] Shimaoka Y, Hidaka Y, Tada H (2000). Changes in cytokine production during and after normal pregnancy. Am J Reprod Immunol.

[29] Matthiesen L, Ekerfelt C, Berg G, Ernerudh J (1998). Increased numbers of circulating interferon-?- and interleukin-4-secreting cells during normal pregnancy. Am J Reprod Immunol.

[30] Wegmann T, Lin H, Guilbert L, Mosmann T (1993). Bidirectional cytokine interactions in the maternal-fetal relationship: is successful pregnancy a TH2 phenomenon?. Immunol Today.

[31] Marvin K, Keelan J, Eykholt R, Sato T, Mitchell M (2002). Use of cDNA array to generate differential expression profiles for inflammatory genes in human gestational membranes delivered at term and preterm. Mol Hum Reprod.

[32] Cooper AM, Khader SA (2007). IL-12p40: an inherently agonistic cytokine. Trends Immunol.

[33] Yoon BH, Romero R, Kim CJ, Jun JK, Gomez R, Choi JH (1995). Amniotic fluid interleukin-6: a sensitive test for antenatal diagnosis of acute inflammatory lesions of preterm placenta and prediction of perinatal morbidity. Am J Obstet Gynecol.

[34] Hollier LM, Rivera MK, Henninger E, Gilstrap LC, Marshall GD (2004). T helper cell cytokine profiles in preterm labor. Am J Reprod Immunol.

[35] Turra SE, Damaso EL, Veiga EC, Cardoso VC, Bettiol H, Cavalli RC (2023). Serum cytokines in second trimester pregnancy and their relationship with spontaneous preterm births in the Ribeirão Preto and São Luiz cohorts. BMC Pregnancy Childbirth.

[36] Curry A, Vogel I, Drews C, Schendel D, Skogstrand K, Flanders W (2007). Mid-pregnancy maternal plasma levels of interleukin 2, 6, and 12, tumor necrosis factor-alpha, interferon-gamma, and granulocyte-macrophage colony-stimulating factor and spontaneous preterm delivery. Acta Obstet Gynecol Scand.

